# Intake of 25-hydroxyvitamin D3 reduces duration and severity of upper respiratory tract infection: A randomized, double-blind, placebo-controlled, parallel group comparison study

**DOI:** 10.1007/s12603-017-0952-x

**Published:** 2017-07-21

**Authors:** Yoshiki Shimizu, Y. Ito, K. Yui, K. Egawa, H. Orimo

**Affiliations:** 1 FANCL Research Institute, FANCL Corporation, 12-13 Kamishinano, Totsuka-ku, Yokohama, Kanagawa, 244-0806 Japan; 2 Nihonbashi Egawa Clinic, Tokyo, Japan; 3 Japan Osteoporosis Foundation, Tokyo, Japan

**Keywords:** 25-hydroxyvitamin D3, 25-hydroxycholecalciferol, calcifediol, vitamin D3, upper respiratory tract infection, common cold

## Abstract

**Objectives:**

This study aimed to assess the effect of 25-hydroxyvitamin D3 (25OHD) which is a hydroxide of vitamin D3 ingestion on upper respiratory tract infection (URTI).

**Design and Setting:**

A prospective, randomized, double-blind, placebo-controlled study was performed from December 2015 to September 2016 in the Nihonbashi Egawa Clinic, Kei Medical Office TOC Building Medical Clinic, and Medical Corporation Kaiseikai Kita-Shinyokohama Medical Clinic, in Japan.

**Participants:**

Four hundred twenty eight participants aged 45-74 years were screened by their serum 25-hydoroxyvitamin D concentration.

**Intervention:**

The participants were randomized to either 25OHD (10 μg/day) or placebo capsule, daily, for 16 consecutive weeks.

**Measurements:**

The primary outcome measure was the incidence proportion of URTI, and the secondary outcome measures were the physical severity score, the quality-of-life (QOL) score, the duration of URTI, and the incidence proportion of new URTI events every four weeks. Data were collected using cold diary Wisconsin Upper Respiratory Symptom Survey-21 (WURSS-21) during the intervention.

**Results:**

Of 428 participants screened, 252 with serum 25-hydroxyvitamn D levels were deficient or insufficient (75 nmol/L or less) were enrolled in this study. Of these, 105 placebo and 110 25OHD group subjects completed the study. For the incidence proportion of URTI, no effect of 25OHD intake was observed. On the other hand, the duration of URTI was shorter in the 25OHD (P = 0.061) compared to placebo. For the incidence proportion of URTI every four weeks, the incidence of new URTI was decreased in both groups over the time of intake. However, when the 25OHD and the placebo were compared, a decrease in the incidence proportion of URTI was seen earlier in the 25OHD. When the total physical severity score and the total QOL score during the study were assessed, they both were significantly improved in the 25OHD compared to placebo.

**Conclusions:**

The intake of 25OHD may reduce the duration of URTI, the physical severity, and the QOL when suffering from URTI.

## Introduction

In general, the common cold is a group of diseases that present with various upper respiratory tract symptoms, including runny nose, nasal congestion, sneezing, sore throat, and coughing, and is a group of diseases caused by many different types of viruses and bacteria. Normally, the common cold remits spontaneously without treatment (1). The average adult gets two to four colds a year, while the average child may get six to eight. In the United States, it is assumed that people are absent from school 22 million days and from work 20 million days in total due to a cold, and there is a report that the economic loss caused by colds amounts to 25 billion US dollars a year (1, 2).

Due to the fact that causes of the common cold are associated with many different types of viruses and bacteria, vaccination is not practical as a preventive measure. For this reason, symptomatic treatment is the main measure for colds.

Recent studies have reported that vitamin D affects various components of the immune system. For example, antimicrobial peptide LL-37, which is considered to be important for innate immunity, is a peptide produced by cleavage of human cathelicidin by proteinase 3, and has high antibacterial activity and antiviral activity (3-6). It has been reported that the expression level of LL-37 is increased by vitamin D and that the serum LL-37 level is low when the serum 25-hydroxyvitamin D level is low (7, 8). It has also been reported that the plasma LL-37 level is increased by the intake of vitamin D3 (9). Based on these reports, it is considered that LL-37 is involved in the protective effect of vitamin D intake against respiratory tract infection.

There have been several studies on vitamin D in the blood and respiratory tract infection, and the relationship between the serum vitamin D level and the incidence of respiratory tract infection has been reported (10, 11). From these findings, the effects of vitamin D intake on respiratory tract infection have been studied. In fact, it has been reported in RCTs and meta-analyses that vitamin D intake reduces the incidence of respiratory tract infection (12-15).

Vitamin D in humans is sourced from the synthesis in the skin through exposure to UV light and from intake of food containing vitamin D2 or vitamin D3 such as fish, dairy products, and mushrooms. Synthesized or ingested vitamin D is mainly hydroxylated in the liver at position 25 by CYP2R1 or CYP27A1 that belongs to the cytochrome P450 superfamily, and converted to 25-hydroxyvitamin D (16).

25-hydroxyvitamin D binds to vitamin D-binding protein, and circulates in the bloodstream. Subsequently, 25-hydroxyvitamin D transported to the kidneys is hydroxylated in a cell at the 1α position by CYP27B1 to form 1α, 25 (OH)2 vitamin D (17, 18). 1α, 25 (OH)2 vitamin D, an active form of vitamin D, acts by binding to organs, tissues, and immunocompetent cells where vitamin D receptors are expressed (19-21). Since CYP27B1 is expressed also in bronchial epithelial cells and hydroxylation of 25-hydroxyvitamin D is possible, it is considered that 1α, 25 (OH)2 vitamin D is important for early pathogen defense (22).

Although studies and applications of vitamin D and 1α, 25 (OH)2 vitamin D3 derivatives have been accomplished, the application of 25-hydroxyvitamin D3 (25OHD) has not been advanced. Regarding the relationship between serum 25-hydroxyvitamin D level and lower limb muscle strength, Bischoff-Ferrari et al. have reported that 25OHD taken by postmenopausal Caucasian women has a reinforcement effect on lower limb muscle strength (23, 24). In addition, ingestion of 25 OHD and exercise improved survival after surgery for an osteoporotic hip fracture (25). On the other hand, the effect of 25OHD intake on immune function in humans is uncertain.

Hence, in this study, the effect of 25OHD intake on upper respiratory tract infection (URTI) was assessed using placebo as a control.

## Subjects and Methods

Study implementation: This study was conducted after it was reviewed and approved (approval date: December 11, 2015) by the HUMA R&D Ethical Review Committee consisting of third parties who have no involvement in the study. In accordance with the spirit of the Declaration of Helsinki (originally adopted in June 1964, and revised in October 2013), the study was implemented within the Ethical Guidelines for Medical and Health Research involving Human Subjects (Public Notice of the Ministry of Education, Culture, Sports, Science and Technology and the Ministry of Health, Labour and Welfare No. 3 of 2014). (UMIN ID: UMIN000020169)

The purpose and content of the study were fully explained by the principal investigator to the eligible subjects, and the study was performed only on those who gave consent in writing on their own free will.

For this study, the assessment of physical findings, physical measurements, physical examinations, and clinical examinations were conducted at Nihonbashi Egawa Clinic, Kei Medical Office TOC Building Medical Clinic, and Medical Corporation Kaiseikai Kita-Shinyokohama Medical Clinic, where the study subjects were managed and the study implementation system was maintained. The principal investigator controlled the operations related to the study; instructions and explanations to the study subjects, acquisition of written informed consent from the subjects, medical interviews, confirmation and assessment of adverse events, and maintenance of the study implementation system were all performed by the principal investigator. We also planned to give treatment to adverse events as needed. This study was implemented between December 2015 and September 2016.

### Subjects

Background surveys including medical history, drinking habit, and eating habit, physical condition checkup, measurements, and clinical examinations were performed as a screening test on the 428 subject candidates who gave written consent, and 252 cases, who met the selection requirements and did not meet the exclusion criteria, were selected as the study subjects.

For the selection of study subjects, those who had the capacity to consent to the study, who met the following: I. Healthy Japanese between 45 and 74 years of age at the time of consent, II. Subject whose serum 25-hydroxyvitamin D level is 30 ng/mL (= 75nmol/L) or less, and III. Subject whose BMI is between 18.5 kg/m2 and 24.9 kg/m^2^, and who did not meet the following exclusion criteria, were selected. I. Current smoker, II. Subject with severe disease (e.g., liver disease, renal disease, infectious disease, cancer), III. Subject with disease that would affect the study results (e.g., hypercalcemia), IV. Subject who has a plan to be exposed excessively to the sun (e.g., farm work, vacation in resorts) during the study, V. Subject whose fasting glucose level is 110 mg/dL or more, VI. Subject who received hormone replacement therapy for the past six months, VII. Subject who would take other supplements than multivitamins during the study; this excludes person who will stop taking them before the study and will not take any until the study ends, VIII. Subject who took vitamin D supplements for the past three months, IX. Subject who took 600 mg/day or more of calcium supplement for the past three months, X. Subject with hypertension: the systolic blood pressure at rest is 145 mmHg or higher, or the diastolic blood pressure is 95 mmHg or higher, XI. Subject being treated for hypertension, XII. Subject with history of mental illness, person in a state where it is difficult to understand the study content, XIII. Subject who does a highintensity exercise continuously (who does a high-intensity exercise more than three times a week), XIV. Subject in a state that would affect the absorption of the trial supplement: impairment of intestinal absorption, sprue (syndrome with impairment of small-intestinal absorption), colitis, gastroenterological surgery, M. Whipple disease (respiratory insufficiency syndrome due to systemic bacterial infection), XV. Subject with disease that carries a risk of hypercalcemia: multiple organ granulomatous disease (sarcoidosis), tuberculosis, lymphoma, primary hyperparathyroidism, XVI. Subject with kidney stones, XVII. Subject whose creatinine clearance is 30 mL/min or less (severe renal failure), XVIII. Subject who is sensitive or allergic to dairy products, XIX. Subject using drugs: anticoagulants, steroids, parathyroid hormone, thiazide diuretics, anticonvulsants, antipsychotics, drugs for schizophrenia, drugs that affect fat absorption (peripheral anti-obesity drugs such as Xenical and Alli, fat absorption inhibitors), XX. Subject who used drugs that could affect bone metabolism (Bisphosphonate, hormone replacement therapy drugs, estrogen-receptor regulators, calcitonin) for the past one year, XXI. Subject who consumes a non-fat diet or has other extreme dietary habits, XXII. Subject who is on a weightloss program or whose diet is under the supervision of a doctor, XXIII. Subject with symptoms of acute or severe diseases: unintended weight loss, night sweats, and cancer treatment, XXIV. Subject whose alcohol consumption is 140 g/week or more, and XXV. Subject who was judged to be unsuitable for the study by the principal investigator or other investigators.

### Trial Supplement

For the trial supplement, a hard capsule formulation containing 10 μg of 25-hydroxivitamin D3 (25OHD) was used. Placebo was prepared by replacing 25OHD with crystalline cellulose so that it would not be distinguished from the trial supplement by the color. 25OHD was provided for free by DSM Nutritional Products, Ltd. (Heerlen, Netherlands).

### Upper respiratory tract symptom survey

For the assessments of the incidence of URTI, the physical severity, and the quality-of-life (QOL), the Japanese version of Wisconsin Upper Respiratory Symptom Survey-21 (WURSS- 21) was used (26). WURSS-21 consists of 21 questions, and the first question (0-7 Likert scale: 0 = Not sick, 1 = Very mildly, 3 = Mildly, 5 = Moderately, 7 = Severely) was answered by the study subjects every day during the study to determine the onset and the duration of URTI.

The next 10 questions (the second to the 11th) are questions about 10 physical symptoms of URTI (Runny nose, Plugged nose, Sneezing, Sore throat, Scratchy throat, Cough, Hoarseness, Head congestion, Chest congestion, and Feeling tired), and they were answered on a 0-7 Likert scale (0 = Do not have this symptom, 1 = Very mild, 3 = Mild, 5 = Moderate, and 7 = Severe) to obtain the total score as the physical severity score. The next nine questions (the 12th to the 20th) are questions about nine events associated with QOL (Think clearly, Sleep well, Breathe easily, Walk, climb stairs, exercise, Accomplish daily activities, Work outside the home, Work inside the home, Interact with others, and Live your personal life), and they were answered on a 0-7 Likert scale (0 = Not at all, 1 = Very mildly, 3 = Mildly, 5 = Moderately, and 7 = Severely) to obtain the total score as the QOL score which is reflected mental severity.

The date of occurrence of URTI was the day on which the subject selected 1-7 after selecting 0 two days in a row for the first question, and the date of disappearance was the day, which was two days prior to the time when the subject selected 0 two days in a row after the onset (the day 1-7 was selected last). The events of URTI in this study were the ones whose duration (the period from the date of occurrence to the date of disappearance) was two days or more, and whose highest physical severity score when suffering from URTI was over 10. For the duration of URTI, the physical severity score, and the QOL score, the highest values during the study were evaluated.

### Trial protocol

The study was a randomized, double-blind, placebocontrolled, parallel group comparison study. Hence, a staff who has no involvement in the study prepared an assignment list using random numbers, and after the study subjects were randomly divided into the 25OHD group and the placebo group, 25OHD and placebo were given to the two groups respectively. The subjects for blinding were all those who were involved in the study, and the assignment was unblinded after securing analysis subjects

During the study, the subjects were required to fill in the WURSS-21 and to record subjective symptoms and whether they made a deviation from instruction such as intake of medical products and health food in their journals. The duration of the supplement intake of this study was from winter (January 2016) to spring (May 2016).

A body measurement, a blood pressure measurement, a pulse measurement, clinical examinations, and the food frequency questionnaire (FFQg ver. 4.0 (Kenpakusha, Tokyo, Japan)) were performed at baseline and 16-weeks tests (27). For the clinical examinations, as a blood test, 25-hydroxyvitamin D, 1α,25 (OH)2 vitamin D, serum Ca, intact parathyroid hormone (PTH) were measured, and as a urine test, urine calcium, and urine creatinine were measured. The clinical examinations were performed by a standard method at SRL Inc. (Tokyo, Japan).

Clinical laboratory tests were conducted using a γ-counter, ARC 950 (Hitachi, Ltd., Tokyo, Japan), Modular Analytics (Hitachi, Ltd., Tokyo, Japan), and clinical chemistry analyzer, BioMajesty ™ series JCA-BM 8060 (JEOL Ltd., Tokyo, Japan). For 25-hydroxyvitamin D, the 25-hydroxy-vitamin D 125I RIA Kit (DiaSorin S. P. A, Saluggia, Italy) based on the RIA 2 antibody method was used. The CV% of the 25-hydroxyvitamin D measurement during the test period was in the range of 8.8% to 12.1%.

### Outcome measures

Primary outcome measure: The primary outcome measure was the incidence proportion of URTI for the period of 16 weeks of supplement intake. The incidence proportion of URTI was calculated by dividing the number of study subjects who developed URTI in each group by the number of study subjects in each group.

Secondary outcome measures: The secondary outcome measures were the physical severity score, the QOL score, the duration of URTI, and the incidence of new URTI every four weeks. For the physical severity score, the QOL, and the duration of URTI, the study subjects who developed URTI were assessed.

### Subgroup analysis

The guideline by the task team of Michael F. Holick et al. says that the risk of osteomalacia and rickets is increased in a deficiency state where the serum 25-hydroxyvitamin D level is 50 nmol/L (= 20 ng/mL) or lower, and that the state where the serum 25-hydroxyvitamin D level is between 50 nmol/mL and 75 nmol/L (= 30 ng/mL) is an insufficiency state where the risk of falling is increased (28, 29). Based on the report that a decrease in the serum 25-hydroxyvitamin D level increases the incidence of URTI, the incidence of URTI, the physical severity score, the QOL score, the duration of URTI, and the incidence of new URTI every four weeks were assessed in the study subjects whose serum 25-hydroxyvitamin D levels at the test before the supplement intake were in a deficiency state and in an insufficiency state respectively.

### Exploratory efficacy analysis

As an exploratory efficacy analysis not listed in the protocol, the total physical severity score and the total QOL score were assessed. Each total score was calculated by adding all the scores when suffering from URTI during the supplement intake.

### Safety assessment

The safety assessment was conducted based on changes in laboratory values, complaints of symptoms from the study subjects, and conventional assessments for adverse events by a doctor.

### Study population

The efficacy analysis was conducted on the per-protocol set (PPS) who fulfilled the protocol. The PPS was a group that excluded out of the full analysis set (FAS): I. Person whose intake rate of the trial supplement was below 80%, II. Person who had developed URTI on the start date of supplement intake, and III. Person who was judged by the principal investigator to be appropriate for being excluded from the study.

The safety assessment was conducted on the FAS.

### Statistical analysis

The primary outcome measure, the incidence proportion of URTI during the period of 16 weeks of supplement intake, and a secondary outcome measure, the incidence proportion of new URTI every four weeks were assessed by Fisher’s exact test. For assessments of the other secondary outcome measures, the generalized Wilcoxon test was used for the duration of URTI, Student’s t test was used for the physical severity score and the QOL score.

The significance level of the study was set to 5% on both sides, and 10% on both sides were marginally significant. JMP® 12 (SAS Institute Inc., Cary, North Carolina, USA) was used for all the analyses.

## Results

### Subject background characteristics at baseline

**Figure 1:**
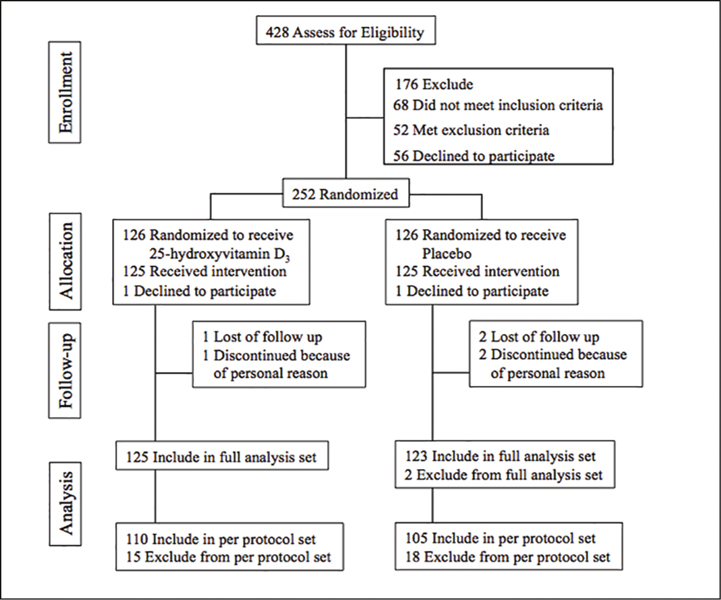
Flow diagram of study participants

The flowchart of this study is shown in Figure 1.

A screening test was performed on the 428 subject candidates who gave consent, and 252 subjects were selected. These 252 cases were randomly assigned to the 25OHD (126 cases) and the placebo (126 cases). Among them, two cases (one case in the 25OHD and one case in the placebo) withdrew their consent before taking the trial supplement and dropped out. As a result, 250 cases started taking the trial supplement. During the intake of the trial supplement, one case in the 25OHD and two cases in the placebo withdrew their consent for the reason that they could not come to the final test due to personal reasons, and dropped out. Therefore, 124 cases in the 25OHD and 123 cases in the placebo completed the predetermined study schedule. Out of the subjects included in the study, one case in the placebo group, whose credibility on the efficacy evaluation and the intake of the trial supplement was questioned based on the responses to the dietary survey, and one case in the placebo, who turned out to meet the exclusion criteria because he had started treatment of hypertension before the supplement intake, were excluded from the FAS, and therefore, the FAS had 125 cases in the 25OHD and 123 cases in the placebo. Among the FAS, out of the subjects who completed the study schedule, one case in the 25OHD and one case in the placebo, who did not comply with the restrictions, and 13 cases in the 25OHD and 15 cases in the placebo, who had developed URTI on the start date of supplement intake, were excluded from the PPS. Therefore, 110 cases in the 25OHD and 105 cases in the placebo were in the PPS. The intake rate of the trial supplement was 99.8% in the 25OHD and 99.4% in the placebo. The number of subjects for safety assessment was 248 cases in the FAS (125 cases in the 25OHD, 123 cases in the placebo).

The background information about the study subjects in the PPS in this study is shown in Table 1.

**Table 1:**
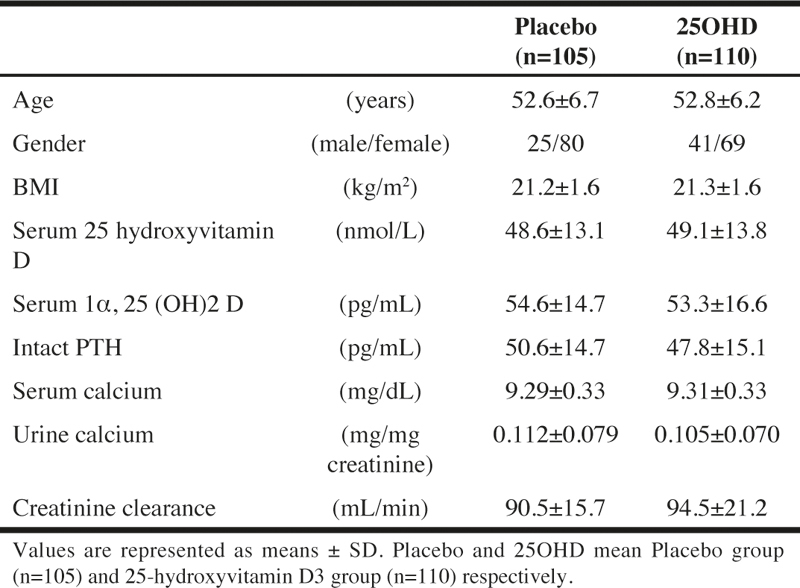
Study subject baseline characteristics

For the serum 25-hydroxyvitamin D levels, the subjects who were determined to be in the deficiency state (50 nmol/L or less) were 61 subjects in the 25OHD and 60 subjects in the placebo, and the subjects who were in the insufficiency state (between 50 nmol/L and 75 nmol/L) were 49 subjects in the 25OHD and 45 subjects in the placebo; the difference was not significant between the two groups (P = 0.891).

The results of the dietary survey are shown in Table 2.

There was no item that significantly changed during the period of 16 weeks of the trial supplement intake in the 25OHD or in the placebo, and there was also no item that showed a significant difference between the two groups. Hence, it was speculated that there was no great change in dietary habits during this study.

### Efficacy evaluation

#### Primary outcome measure

In this study, the number of subjects who developed URTI during the supplement intake was 41 subjects in the 25OHD and 43 subjects in the placebo respectively. The total number of URTI events during the intake was 52 cases in the 25OHD and 66 cases in the placebo respectively.

The incidence of URTI during the period of 16 weeks of supplement intake, which was the primary outcome measure, was 37.3% in the 25OHD and 41.0% in the placebo, and no statistically significant difference was observed between the two groups (P = 0.675). (Table 3)

#### Secondary outcome measures

The physical severity score was 26.7 ± 11.6 in the 25OHD and 31.2 ± 16.3 in the placebo, and although it was lower in the 25OHD, no statistically significant difference was observed (P = 0.154). The QOL score was 27.6 ± 17.2 in the 25OHD and 31.3 ± 19.3 in the placebo, and although it was lower in the 25OHD, no statistically significant difference was observed (P = 0.346).

For the duration of URTI, the 25OHD had lower numbers (median: 10 days) compared to the placebo (median: 13 days) (P = 0.061) (Table 3).

Because 25-hydoxyvitamin D activation (hydroxylation) occurs mainly in the kidneys, an analysis of the duration of URTI was performed with creatinine clearance and gender, which are renal function markers, as adjustment factors. As a result, the supplement effect was significant (P = 0.046), and the shortening effect of the intake of 25OHD on the duration of URTI was suggested.

Although the incidence proportion of new URTI every four weeks decreased both in the 25OHD and in the placebo over the season, no statistically significant difference in the incidence was observed between the two groups. However, the decrease in the incidence occurred earlier in the 25OHD than in the placebo, which was likely the result of 25OHD intake (Table 4).

### Subgroup analysis

The incidence proportion of URTI, the physical severity score, the QOL score, the duration of URTI, and the incidence proportion of new URTI every four weeks in the 25-hydroxyvitamin D deficiency group, whose serum 25-hydroxyvitamin D levels were 50nmol/L or less at the time of screening and in the 25-hydoxyvitamin D insufficiency group, whose serum 25-hydoxyvitamin D levels were between 50 nmol/L and 75 nmol/L, are shown in Tables 3 and 4.

For the incidence of URTI, no significant lowering effect of 25OHD intake on the incidence was observed in the 25-hydroxyvitamin D deficiency group or insufficiency group. However, in the 25OHD, the incidence proportion of URTI was lower in the 25-hydroxyvitamin D deficiency group compared to the insufficiency group, and it was considered that a high serum 25-hydroxyvitamin D level is effective in preventing the onset of URTI.

For the incidence proportion of new URTI every four weeks, a decrease in the incidence was observed both in the D deficiency group and the insufficiency group over the time of the study. However, no significant difference was observed between the 25OHD and the placebo in each group of subjects. Looking at the difference in the incidence proportion of URTI between the 25OHD and the placebo, the difference at 9-12 wk was the greatest (25OHD: 8.2%, placebo: 18.3%, P = 0.115) in the deficiency group. On the other hand, the difference between the 25OHD and the placebo was the greatest (25OHD: 10.2%, placebo: 22.2%, P = 0.159) at 5-8 wk in the insufficiency group, and a decrease in the incidence of URTI in the 25OHD occurred earlier in the insufficiency group than in the deficiency group.

**Table 2:**
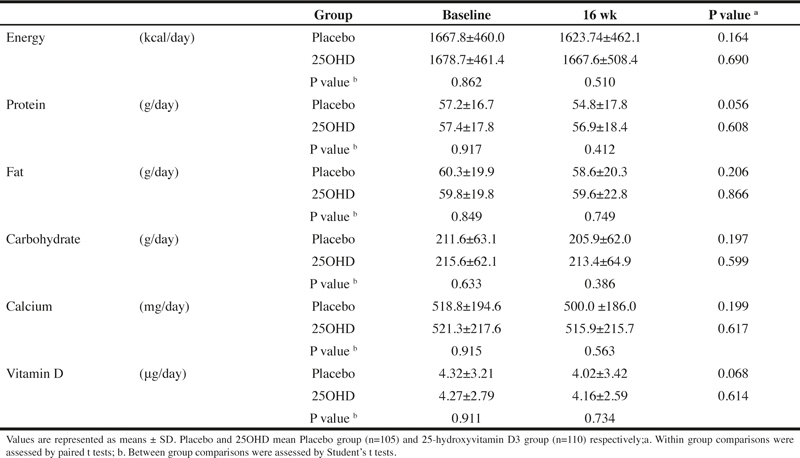
Food frequency questionnaire

For the physical severity score and the QOL score, no statistically significant difference due to the 25OHD intake was observed in the deficiency group and in the insufficiency group. However, in the deficiency group, the QOL score was lower (P = 0.167) compared to the placebo. On the other hand, in the insufficiency group, the physical severity score was lower (P = 0.192) compared to the placebo.

### Exploratory efficacy evaluation

In order to comprehensively evaluate the physical severity of URTI and the decrease in QOL due to URTI, the total physical severity score and the total QOL score during the intake of the trial supplement were calculated respectively. The results are shown in Table 5.

The total physical severity score was significantly lower (P = 0.0496) in the 25OHD compared to the placebo. The total QOL score was significantly lower (P = 0.019) in the 25OHD compared to the placebo. These results suggested that the intake of 25OHD improves the physical severity and the QOL when suffering from URTI.

### Safety assessment

#### Clinical test results

Although significant changes were seen in some items, the principal investigator judged that they all were changes without any clinical significance. For reference, the changes in serum 25-hydroxyvitamin D, 1α, 25 (OH)2 vitamin D, intact PTH, serum calcium, and urinary calcium by the supplement intake are shown in Table 6. No subject developed hypercalcemia during the period.

#### Adverse events

256 adverse events occurred in 73 subjects in the 25OHD, and 339 occurred in 74 subjects in the placebo. The adverse events that occurred include symptoms such as the common cold and a stomachache. Those symptoms were all mild, and they disappeared during the study. For all the adverse events, the principal investigator denied the connection with the trial supplement.

**Table 3:**
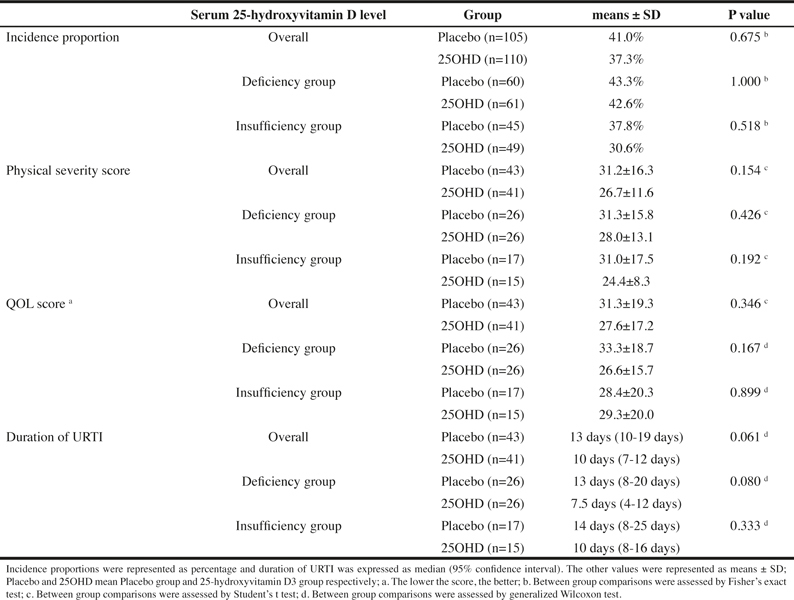
The effects of 25-hydroxyvitamin D3 intake on the incidence of upper respiratory tract infection, the physical severity, the QOL, and the duration of URTI

As seen in the above results, there was no problem in the safety of the trial supplement under the conditions of this study.

## Discussion

URTI is considered to cause serious economic losses, and it is believed that the prevention of this infection and the improvement in symptoms are economically important even from the perspective of public health. Vitamin D3 has been examined for the impact on the protective effect against a cold and influenza, while 25OHD has not been examined. Therefore, in this study, the effect of intake of 25OHD on URTI was examined.

As a result, there was no effect of 25OHD intake observed in the incidence proportion of URTI during the period of 16 weeks of supplement intake, which was the primary outcome measure. However, at the time of 9-12 weeks after the start of intake when it was considered that the serum 25-hydroxyvitamin D level was increasing due to the intake of 25OHD, a considerable difference from the placebo group was seen in the incidence proportion of URTI. This suggests the possibility of preventing URTI by raising the serum 25-hydroxyvitamin D level. This can be explained also by the fact that a decrease in the incidence of URTI was seen earlier in the group of subjects whose serum 25-hydroxyvitamin levels were insufficient compared to the group of subjects whose serum 25-hydroxyvitamin D levels were deficient.

For the duration of URTI, it was suggested that the duration is shortened by 25OHD intake, and it was also suggested that the physical severity and the QOL when suffering from URTI are improved by 25OHD intake.

**Table 4:**
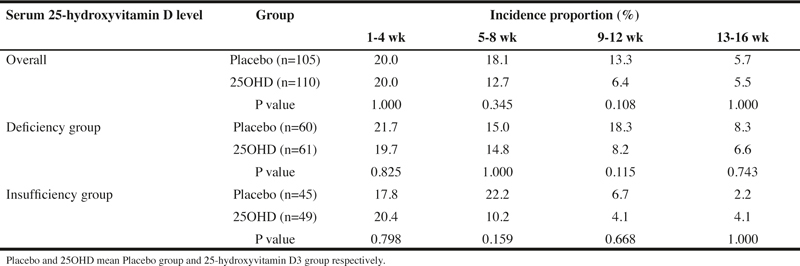
The incidence of new upper respiratory tract infection every four weeks after the start of intake of the trial supplement

The shortening effect on the duration of URTI and the improvement of the physical severity and the QOL, which are thought to be the results of 25OHD intake, can be associated with protection from viruses and bacteria and proliferation inhibition after infection due to 25OHD intake. It is speculated that protection from viruses expected by 25OHD intake is associated with antimicrobial peptide LL37 and secretory immunoglobulin A (sIgA).

**Table 5:**
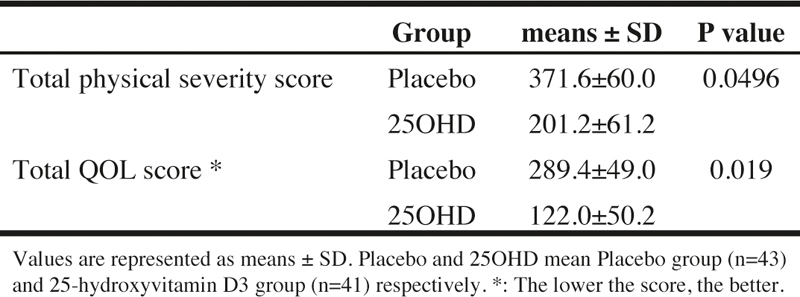
The effects of 25-hydroxyvitamin D3 intake on the total physical severity score and the total QOL score of upper respiratory tract infection

It has been reported that LL37, an antimicrobial peptide whose production is enhanced by 1α, 25(OH)2 vitamin D3, a hydroxylated metabolite of 25OHD, activates TLR3 and TLR9, which are involved mainly in virus recognition among the Tolllike receptors (TLRs), pattern recognition receptors (30, 31). TLR3 and TLR9 activated by 1α, 25 (OH)2 vitamin D3 seem to induce the production of INF-β by a signaling cascade and to have antiviral activity.

In other words, it is assumed that the intake of 25OHD increased the production of LL37 and showed antiviral activity.

The fact that the reduction of symptoms and the shortening of the duration of disease can be expected by suppressing viral growth shows that anti-influenza virus drugs, Oseltamivir and Zanamivir also have effects on the shortening of the duration of influenza and the reduction of severity by suppressing the growth of influenza viruses (32, 33).

In addition, since there is a report that taking Oseltamivir and Zanamivir prophylactically protects against the onset of influenza, suppressing viral growth is considered to not only delay the incidence of URTI but also shorten the duration of URTI and reduce the severity (34-36). In this study, although no decreasing effect of 25OHD intake on the incidence of URTI was observed, it was suggested that the intake of 25OHD shortens the duration of URTI and reduces the severity. It is possible that with 25OHD intake, the antiviral effect induced by antimicrobial peptides such as LL37 contributes to the above-mentioned effects. Since Vitamin D3 has been reported to suppress an inflammatory response, it is speculated that the reduction of the severity of URTI is associated with the antiinflammatory effect of 25OHD (37).

It has been reported that there is a correlation between serum 25OHD and salivary sIgA, and salivary sIgA is expected to increase by way of raising the serum 25-hydroxyvitamin D level (9). In fact, it has been reported that salivary sIgA increases when 5000 IU of vitamin D3 is taken daily for 14 weeks (10). It is possible that the intake of 25OHD, as with the case of vitamin D3, could increase sIgA.

It is believed that these presumed functions of 25OHD are exerted when 25OHD is hydroxylated by CYP27B1. However, since the blood level of 25OHD is about 1000 times higher than the blood level of its hydroxide, 1α,25 (OH)2 vitamin D, 25OHD itself may also be exerting the functionality.

The influence of vitamin D intake on respiratory infection (RTI) has evaluated by meta-analysis. Several meta-analyses have demonstrated the reduction of the incidence and shortening the duration of RTI by intake of vitamin D (13-15).

**Table 6:**
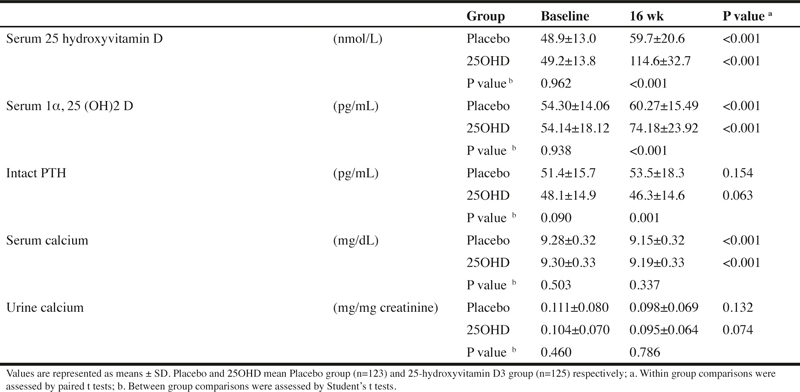
Serum vitamin D and calcium metabolism-related items

Meanwhile, in meta-analysis by Vuichard Gysin D et al., there is no preventive effect of RTI or shortening of the duration of disease by ingestion of vitamin D in healthy subjects (38). Such differences in conclusions are thought to be due to the difference in the dosage, intake method (daily or bolus ingestions) and intake period of vitamin D. Therefore, we believe that the results of this study are not to be denied. Also, Intake of 25 OHD may better evaluate the effect on URTI by improving study design.

The result of this study suggests that the intake of 25OHD will be a new option to reduce the burden of URTI with respect to physical severity and QOL and to accelerate the healing of URTI.

### Limitations

There are some research limitations in this study. First, it seems that the number of cases in this study was too small to statistically significantly prove the efficacy of 25OHD intake. Secondly, immune markers such as antimicrobial peptides and salivary sIgA are not measured. Thirdly, because the epidemic of URTI is dependent upon the season, it is difficult to distinguish it from the effects of 25OHD intake. Fourthly, since the clinical examinations were conducted only twice: before the intake and after the intake, the behavior during the intake could not be assessed. Fifthly, the evaluation of URTI may be biased, because subjects recorded their subjective symptoms of URTI using WURSS-21.

Based on the above facts, it is considered that there is a need to conduct a study with larger number of samples, measure immune markers, laboratory examination, and conduct examinations at multiple points of time in the future.

*Conflict of interest:* This study was conducted with funds from FANCL Corporation (FANCL). 25OHD used in this study was provided for free by DSM Nutritional Products, Ltd. YS, YI, and KY are employees of FANCL. Based on the results of this study, FANCL has applied for a patent. YS and YI are inventors in patent law. FANCL has paid a supervision fee to HO. There are no other conflicts of interest to be noted.

*Acknowledgment:* We would like to express sincere gratitude to the 25OHD3 research group established in Medical Corporation Foundation Kenkoin Clinic (Tokyo, Japan) who provided valuable advice for preparing this paper. YS, YI, KY, and HO designed this study; KE conducted research; YS analyzed data; and YS, YI, KY and HO wrote the paper. YS had primary responsibility for final content. All authors read and approved the final manuscript.

*Ethical standard:* This study was conducted according to the spirit of the Declaration of Helsinki (originally adopted in June 1964, and revised in October 2013) and the Ethical Guidelines for Medical and Health Research involving Human Subjects (Public Notice of the Ministry of Education, Culture, Sports, Science and Technology and the Ministry of Health, Labour and Welfare No. 3 of 2014). All procedures were approved by the HUMA R&D Ethical Review Committee. Written informed consent was obtained from all participants. No animals were used in this study.

## Abbreviations

25OHD: 25-hydroxyvitamin D3
URTI: upper respiratory tract infection
QOL: quality-oflife
WURSS-21: Wisconsin Upper Respiratory Symptom Survey-21
BMI: body mass index
sIgA: secretory immunoglobulin A
PTH: parathyroid hormone
